# A WKNN Indoor Fingerprint Localization Technique Based on Improved Discrimination Capability of RSS Similarity †

**DOI:** 10.3390/s24144586

**Published:** 2024-07-15

**Authors:** Baofeng Wang, Qinghai Li, Jia Liu, Zumin Wang, Qiudong Yu, Rui Liang

**Affiliations:** 1 School of Information Technology Engineering, Tianjin University of Technology and Education, Tianjin 300222, China; wangbaofeng@tute.edu.cn (B.W.); 0303180108@tute.edu.cn (Q.L.); yuqiudong@eyou.com (Q.Y.); lrcalr@163.com (R.L.); 2 School of Information Technology Engineering, Hebei University of Environmental Engineering, Qinhuangdao 066102, China; 3 College of Information Engineering, Dalian University, Dalian 116600, China; wangzumin@163.com

**Keywords:** indoor fingerprint localization, WKNN, fingerprint distances, APs selection, RSS similarity

## Abstract

There are various indoor fingerprint localization techniques utilizing the similarity of received signal strength (RSS) to discriminate the similarity of positions. However, due to the varied states of different wireless access points (APs), each AP’s contribution to RSS similarity varies, which affects the accuracy of localization. In our study, we analyzed several critical causes that affect APs’ contribution, including APs’ health states and APs’ positions. Inspired by these insights, for a large-scale indoor space with ubiquitous APs, a threshold was set for all sample RSS to eliminate the abnormal APs dynamically, a correction quantity for each RSS was provided by the distance between the AP and the sample position to emphasize closer APs, and a priority weight was designed by RSS differences (RSSD) to further optimize the capability of fingerprint distances (FDs, the Euclidean distance of RSS) to discriminate physical distance (PDs, the Euclidean distance of positions). Integrating the above policies for the classical WKNN algorithm, a new indoor fingerprint localization technique is redefined, referred to as FDs’ discrimination capability improvement WKNN (FDDC-WKNN). Our simulation results showed that the correlation and consistency between FDs and PDs are well improved, with the strong correlation increasing from 0 to 76% and the high consistency increasing from 26% to 99%, which confirms that the proposed policies can greatly enhance the discrimination capabilities of RSS similarity. We also found that abnormal APs can cause significant impact on FDs discrimination capability. Further, by implementing the FDDC-WKNN algorithm in experiments, we obtained the optimal K value in both the simulation scene and real library scene, under which the mean errors have been reduced from 2.2732 m to 1.2290 m and from 4.0489 m to 2.4320 m, respectively. In addition, compared to not using the FDDC-WKNN, the cumulative distribution function (CDF) of the localization errors curve converged faster and the error fluctuation was smaller, which demonstrates the FDDC-WKNN having stronger robustness and more stable localization performance.

## 1. Introduction

With the rapid development of wireless communication technology in pervasive infrastructure and mobile clients, the demand for LBSs (location-based services) is also accelerating in people’s daily lives. Hence, indoor localization technologies have gained great concern in both academia and industry, and various indoor localization technologies and techniques have been developed [1,2]. In particular, because of the advantages of no need for additional infrastructure and low cost, indoor WLAN (Wireless Local Area Network)-based localization has become one of the most popular technologies [3], among which fingerprint-based localization technique is one of the hot research topics. The fingerprint-based indoor localization is usually conducted in two phases, namely, the offline site survey phase and the online fingerprint matching phase.

So far, a lot of study work and achievements have been made in these two phases, and many systems have been developed to reduce labor costs and localization errors and improve localization accuracy [4,5]. In many existing indoor fingerprint localization techniques, we found that the similarity of positions is usually discriminated by comparing the similarity of RSS vectors and then used to estimate the coordinates of test points (TPs). For example, multidimensional scaling (MDS) technology is a data dimensionality reduction method widely applied in indoor localization. It often employed FDs to represent PDs to construct a distance matrix between nodes, thereby obtaining their similarity and computing the estimate of the TP [6]. Also, there are a large number of machine learning algorithms that have been introduced into indoor fingerprint localization, many of which utilize FDs to get the estimated coordinate of TP [5,7].

That is to say, using the RSS vector distances (i.e., FDs) between two position points to discriminate the PD between them is a prerequisite and foundation for many fingerprint-based indoor localization techniques. Inspired by this viewpoint, we infer that if the discrimination capability of FDs for PDs is improved, the corresponding localization techniques will also be improved. However, most existing efforts mainly focus on improving fingerprint matching algorithms; the fundamental reasons for the impact of each AP on FD’s capability to discriminate PD have not been fully studied, such as the healthy states of APs and the positions of APs.

It is well known that indoor fingerprint-based localization can fully utilize public WLAN facilities but does not require the accurate locations of the APs. However, with the rapid development and popularization of WLAN infrastructure and smart devices, APs are widely deployed. Our intelligent terminal can easily receive high-quality signals from a large number of APs. The states of these APs are dynamic, with some appearing, some disappearing, some blocked, some malfunctioning, and so on. We refer to all of these situations as the healthy states of the APs. From the perspective of signal strength, the healthy states of APs can be divided into three categories: normal, slightly unhealthy, and complete failure. The latter two are also known as abnormal APs. Due to APs being ubiquitous, we still have sufficient normal APs to use. Meanwhile, in large indoor spaces, the set of APs that can be received varies with the TP’s position. So, in large-scale indoor spaces with a large number of APs, using fingerprints formed by RSSs from all APs not only increases fingerprint dimensions and is time-consuming but also greatly reduces localization accuracy due to dynamic APs’ states.

Although there have been some studies on the issue of AP selection, the AP selection criteria and models hardly have universality due to the complexity of indoor environments and applications [8]. A prevalent AP selection method is to choose APs with the max RSS or mean RSS. However, since RSS similarity is the comparison of RSS vectors between two positions and the APs’ states are dynamic, it can only be used when the AP’s RSS values in both positions are normal; otherwise, this methodology may lead to the possible amplification of the error [9]. By collecting data in the same position at different times and performing post-processing on them, unstable APs can also be found and eliminated, but it is time-consuming and costly [10]. Correspondingly, based on the large number of APs present in indoor spaces, our proposed strategy does not require repeated sampling. Similarly, the AP selection algorithm based on loss rate requires scanning the channel multiple times during the sampling process to select the APs with low loss rate [11], which is also time-consuming. In addition, the APs selection strategy can also be based on mutual information [12], nonuniform quantization RSSI entropy [13], information gain, joint information gain [14], etc. The aforementioned APs selection strategies are mainly determined by analyzing the received RSS, either from a probabilistic or informational perspective, but they are all static. In real indoor scenarios, when comparing the similarity between two positions, it is common that an AP may not be suitable for two positions but may be suitable for the other two. Therefore, the selection of AP subsets should be dynamic, that is, different TPs may select different AP subsets to estimate the coordinates during the online phase.

For the positions of the APs problem, researchers in [15] identified several crucial problems that caused the localization errors, including APs’ discrimination for fingerprinting a specific location and the transitional fingerprint problem on smartphones. However, the impact of APs on RSS similarity of location pairs has not been deeply studied, and the health states of APs have not been taken into account. For APs’ health state problem, in [16], the problem of the impaired APs and the joining of the new APs were considered, and a secure fingerprint localization method was proposed for robust variable environments, but it is aimed at specific environments and collaborates by installing reference anchor nodes to update the fingerprint database. This technique, which requires installation facilities, cannot be directly applied in public, large indoor spaces. Although we have analyzed AP’s discrimination capability in [17], no localization algorithm was provided. Hence, the research of [17] was extended in this article, and a new FDDC-WKNN technique is redefined by combining the policies of improving FD’s discrimination capability to PD and WKNN algorithm, which can provide a solution for the aforementioned problem about APs’ status. As for WKNN, it is a simple and classic machine learning algorithm and also a typical algorithm widely used in indoor positioning [10,18]. The existing improved WKNN algorithm usually directly uses RSS similarity to discriminate the similarity of positions, mainly focusing on the weight parameters and the value of K. In recent years, many researchers have begun to combine WKNN with other more complex algorithms [19,20], which not only increases computational complexity but also reduces time efficiency.

The scenes considered in the article are AP-rich large-scale indoor environments. These APs with high-quality signals are installed for communication, they are evenly distributed and dense, but they are not uniformly managed. That is to say, we do not know the state of most of the APs. When we utilize these ubiquitous APs for localization, they will bring greater interference and uncertainty, seriously affecting localization performance. Therefore, the main focus of our study is to select an appropriate subset of normal APs dynamically based on the TPs and adjust their contribution to the discrimination capability of RSS similarity for PDs based on their states.

The main work and contributions of the article can be summarized as follows:

(1) The root causes limiting the discrimination capability of FDs related to APs’ state and position are investigated. We deeply analyzed and recognized three factors that influence the contribution of APs to FD’s discrimination capability, which are ① the distance between the AP and the sample position, ② the AP’s direction to the pairwise positions, and ③ the healthy states of the APs.
(2) For the above three problems, we provided corresponding solution policies to improve FD’s discrimination capability. For the healthy states of the APs, setting a threshold for all sample RSSs at both offline and online stages will eliminate abnormal APs dynamically. For the distance and direction of APs, a discrimination correction quantity and a priority weight were proposed to adjust APs’ contribution to FD’s discrimination capability.
(3) Ultimately, by integrating the solution policies with WKNN, we advanced a redefined indoor localization technique, that is, FDDC-WKNN, which has strong robustness and stability for the state of AP in AP-rich environments without knowing APs’ status.

The rest of the manuscript is organized as follows. Section 2 is the related work on indoor localization; Section 3 introduces the framework of FDDC-WKNN and presents preliminary knowledge and some symbols definitions; and in Section 4, we conduct some observation to find APs’ factors affecting FD’s discrimination capability and provide corresponding solutions. The FDDC-WKNN algorithm process is described in detail in Section 5, and in Section 6, extensive experiments and simulations are executed. At last, the conclusions of the article are presented in Section 7.

## 2. Related Works

With the modernization of production and lifestyle, more work and entertainment activities can be carried out indoors, and most people spend approximately 80% of their daily lives indoors [5]. Outdoors, satellite positioning, and navigation have provided convenience in all aspects of our lives. However, these conveniences cannot be extended to indoor environments. Therefore, a variety of indoor localization technologies and techniques are booming, which will be reviewed in this section. In addition, the APs selection strategies are also briefly reviewed in this section.

Classified by the dependent infrastructure, indoor localization technologies include Wi-Fi [21,22], acoustic signals [23], UWB (Ultra Wide Band) [24], RFID (Radio Frequency Identification) [25], Bluetooth, etc. Recently, LoRa (Long Range) communication extended these wireless technologies and was employed for indoor positioning. In [26], the LoRa technology and its employment for RSSI-based indoor localization in the license-free 2.4 GHz band were studied, and an average localization error below 2.2 m was obtained. Moreover, other techniques based on acoustics, vision, magnetic fields, accelerometers, and so on are also used to estimate indoor locations, but due to the need to install additional equipment, the cost is high, and it is difficult to widely apply [5]. In addition, a variety of other integrated localization technologies have also been proposed. For example, in [27], a location-aware infrastructure is presented, which can give rise to a sensing mechanism that enables infacility crowdsourcing, which can help fingerprint localization services. Due to the similarity of RSS sequences, the assistant nodes were selected in [28] to improve the localization accuracy, and by using an adaptive Kalman filter, the time-of-flight ranging error has been alleviated. Recently, a fingerprint augment framework based on super-resolution (FASR) was proposed in [29] to reduce the cost at the offline phase and ensure localization accuracy. Based on the conversion between fingerprint images and fingerprint data, the framework achieved the fusion of fingerprint augment and super-resolution. A transportable laser range scanner was used to automatically label Wi-Fi scans in [30] to get an accurate indoor fingerprint localization system with no associated data collection.

Classified by the nature of signals to be measured, various localization techniques have been developed based on the signal nature, such as TDOA (Time Difference Of Arrival), TOA (Time Of Arrival), RSS [7], CSI (Channel State Information) [31], etc. Among all the technologies mentioned above, the fingerprint localization technique based on RSS sampling has been widely studied, and with the vigorous development of artificial intelligence (AI) technology, more and more machine learning algorithms are being applied to the two phases of fingerprint localization [32,33], such as K-nearest neighbor (KNN) [34], weighted K-nearest neighbor (WKNN) [19,35], support vector machine (SVM) [36], artificial neural network (ANN) [37,38], and so on.

To improve indoor positioning accuracy and stability, in [35], the WKNN algorithm was improved by fusing the spatial distance and physical distance (PD) of the RSS, while in [19], a combined algorithm based on WKNN and extreme gradient boosting (XGBoost) was proposed for location determination. For other machine learning methods, to deal with high noise in RSSI measurements and ensure high target-localization accuracy, authors in [39] proposed two range-free target-localization schemes on SVR, one is a plain support vector regression (SVR)-based model and the other is a fusion of SVR and Kalman filter (KF), while in [36], based on multi-output least squares SVM regression, authors built a statistical regression model on the RSS dataset to obtain the locality of a mobile device. By using a back-propagation neural network (BPNN) to calculate the similarities between different fingerprints constructed based on absolute RSS values, a high-adaptability indoor localization method was proposed in [38]. In [40], BPNN is used to determine the distances between the TP and each reference point (RP) to mitigate the effect of the fluctuation of RSS. Moreover, the deep neural network (DNN) model is also considered in indoor fingerprint localization [41], and the DNN models obtained from multiple training sessions are combined to locate the target, achieving a lower RMSE. In all, it has been proven that machine learning can effectively improve indoor localization accuracy, enhance system robustness, reduce costs, and improve the performance of the indoor localization method [42]. For Bluetooth Low Energy technology, authors in [32] implemented and analyzed the performance of four supervised learning techniques, that is, KNN, SVM, Random Forest (RF), and ANN, to explore the possible improvement in the localization accuracy and found that the most promising machine learning technique was Random Forest, with classification accuracy over 99%. Furthermore, with the growth of computationally capable smartphones, deep learning has also been used in indoor localization systems [43,44].

Referring to AP selection, some strategies have already been implemented [9]. In the survey by [11], five different AP selection strategies were concluded, and the max mean RSS method and loss rate method were used to perform automatic regulation for the proposed self-adaptive AP selection algorithm. In [13], the authors introduced the nonuniform quantization RSSI entropy (NQRE) to quantify AP’s discernibility and select APs to construct an offline fingerprint database. For the scene where both indoor structure and user moving mode are fixed, an AP selection method was proposed in [45] by adding a perturbation operator to the RSS dataset to extract the correlation between RSSs, which can reduce the time consumption at the online stage. On the contrary, to deploy an optimal AP placement configuration in indoor localization systems, the mean and variance of the RSS were used [46]. Moreover, to effectively select stable and suitable APs, the authors in [47] proposed an indoor localization algorithm based on multiple access point selection, in which two AP selection steps are added at the offline stage. The first step is to delete the APs with less frequency from the RSSI information at each RP, and the second step is using the k-means algorithm to cluster RPs and re-selecting the AP subset for each cluster. However, so far, it is hard to have the universality of AP selection criteria due to the complexity of indoor environments and applications.

## 3. Indoor Fingerprint Localization Model and Preliminary Knowledge

In this section, we first introduce the framework of an indoor fingerprint localization system. Then, some preliminary knowledge and symbol definitions are provided to make the expression clearer later.

### 3.1. The Framework of the Indoor Fingerprint Localization Model

**Figure 1:**
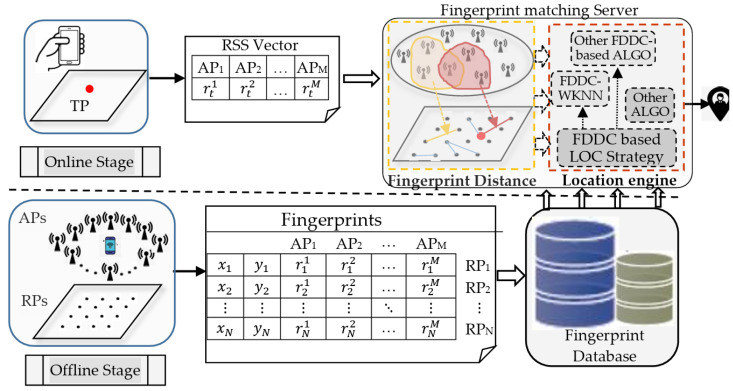
Framework of indoor fingerprint localization system.

The localization process of a typical indoor fingerprint localization technique usually includes two phases, namely, an offline site survey and online fingerprint matching. During the offline phase, for each RP, the RSS values from different APs are collected and recorded as an RSS vector. Add the coordinates of the RP to the RSS vector to form the fingerprint of the RP; then, the fingerprints of all RPs form a fingerprint matrix and are stored in the fingerprint database. During the online phase, a user sends a RSS vector at a TP to the localization server. Through the localization technique based on the fingerprints in the database, the server returns the user’s estimated position (see Figure 1).

Overall, the framework in Figure 1 is consistent with the fingerprint-based indoor localization system. The FDDC-WKNN algorithm proposed in this article is located within the location engine of fingerprint matching sever at the online stage. One of the contributions of this article is to improve the FDs’ discrimination capability (FDDC) for PDs, thereby improving the WKNN localization algorithm. For all other FDDC-based localization algorithms (other FDDC-based ALGO), the FDDC improved strategy presented in this article is also applicable, and they can also be deployed and implemented within the location engine to enhance its localization capability. Therefore, Figure 1 presents the framework structure of a fingerprint indoor positioning system with a scalable location engine.

### 3.2. Preliminary Knowledge

For the convenience of subsequent expression, preliminary knowledge and some important symbol definitions used in the article are described and introduced in this section.

#### 3.2.1. Log-Normal Distance Path Loss Model

Theoretically, RSS decays logarithmically with propagation distance in terms of the propagation law of wireless signals. That is, when the transmitter sends a signal to the receiver, the path loss at the receiver ends logarithmically with the distance between the transmitter and the receiver. Here, to express signal propagation loss in a complex indoor scene, the Log-normal Distance Path Loss model is considered, see Formula (1), and then the received signal strength at the receiver can be obtained by Equation (2) [48].

(1) $$P_{L}\left(d\right)=P_{L}\left(d_{0}\right)+10nlog\left(\frac{d}{d_{0}}\right)+X_{\sigma },$$

(2) $$r\left(d\right)=P_{R}\left(d_{0}\right)-10nlog\left(\frac{d}{d_{0}}\right)+X_{\sigma },$$

Here, all distances are measured in meters. The mathematical symbols and parameters in (1) and (2) are explained as follows:

d: the distance from the transmitter to the receiver.

$r\left(d\right)$: cc receiver, usually measured in decibel-milliwatts(dBm).

$P_{L}\left(d\right)$: the path loss at the receiver.

$d_{0}$: the reference distance, usually 1 m.

$P_{L}(d_{0})$: the path loss at the reference distance.

n: the path loss exponent, that is, the growth rate of path loss with distance, which is based on the different environments and building types, mostly between 2 and 4.

$X_{\sigma }$: Gaussian noise denotes the complex effects of the indoor environment.

$P_{R}\left(d_{0}\right)$: the received signal strength at the reference distance, which is $P_{T}-P_{L}\left(d\right)$ and $P_{T}$ is the transmitter’s transmission power.

#### 3.2.2. The Standard for Measuring the Relationship between Two Vectors

Since utilizing the FD of pairwise positions to discriminate the PD between them is a prerequisite for indoor fingerprint localization technologies, it is necessary to discuss the discrimination capability between two vectors, which means that we need to know under what conditions one vector can be used to represent another vector well. Here, two criteria are considered, that is correlation and consistency. Next, let two vectors be denoted as X and Y, respectively.

- Correlation

In the field of natural sciences, the Pearson correlation coefficient between two variables is widely used to test the degree of correlation, with a value varying from −1 to 1. If the coefficient is between 0.8 and 1.0, the relationship between the two variables is a very strong correlation, and if the coefficient is between 0.6 and 0.8, the relationship between the two variables is a strong correlation. For X and Y, the Pearson correlation coefficient can be calculated by Formula (3) [49]. Then, the coefficient can be used to imply the correlation between FDs and PDs, and it is reasonable use it to indicate the degree of linear correlation between them.

(3) $$\lambda =\frac{\sum_{i=1}^{n}\left(X_{i}-\bar{X})(Y_{i}-\bar{Y}\right)}{\sqrt{\sum_{i=1}^{n}{\left(X_{i}-\bar{X}\right)}^{2}}\sqrt{\sum_{i=1}^{n}{\left(Y_{i}-\bar{Y}\right)}^{2}}},$$

where $\bar{X}$ and $\bar{Y}$ represent the mean values of X and Y, respectively.

- Consistency

Besides, it is reasonable that if the $X_{i}$ is greater than $X_{j}$, the corresponding $Y_{i}$ should also be greater than $Y_{j}$, which can be called the degree of a data sequence. Similarly, if the consistency coefficient is between 0.8 and 1.0, the relationship between the two variables has very high consistency, and if the coefficient is between 0.6 and 0.8, the relationship between the two variables has high consistency. Similar to reference [48], the consistency coefficient of X and Y is defined as follows:

(4) $$c=\frac{\sum_{1<i,j<n,i<j}sign\left[\left(X_{i}-X_{j}\right)*\left(Y_{i}-Y_{j}\right)\right]}{\sum_{1<i,j<n,i<j}1},$$

where

(5) $$sign\left(x\right)=\left\{\begin{array}{cc} 1, & x>0\\ 0, & x\leq 0 \end{array}\right.,$$

From the above formula, there are some unreasonable situations for indoor fingerprint localization techniques. For example, if the PD of two positions is equal while the RSSD is very small, it is reasonable and practical to consider the data sequence between them to be consistent, but it is not consistent with the above formula. In this article, we use the modified formula for consistency coefficient in [17].

The aforementioned double coefficients are mainly used to test the relationships between FDs and PDs. In cases where the relationships have a very strong correlation and very high consistency, by adopting FDs to discriminate PDs in the indoor localization method, not only the uncertainty of signal propagation in the complex indoor environment can be greatly reduced, but also the localization accuracy can be improved and the localization error can be reduced.

### 3.3. Symbols Definitions

For one position i with coordinate $\left(x_{i},y_{i}\right)$, the RSS vector collected from m APs is represented by $R_{i}$ as follows:

(6) $$R_{i}=\left[r_{i}^{1},r_{i}^{2},\ldots ,r_{i}^{m}\right],$$

where $r_{i}^{k},(1\leq k\leq m)$ is the RSS value that is received from the k-th AP. Combining the position coordinates and the RSS vector, we can get the fingerprint $F_{i}$ of the position i:

(7) $$F_{i}=\left[x_{i},y_{i},r_{i}^{1},r_{i}^{2},\ldots ,r_{i}^{m}\right],$$

For the pairwise positions (i,j) consisting of two different positions in the indoor localization area, whose RSSs vectors are $R_{i}$ and $R_{j}$, respectively, and whose coordinates are $\left(x_{i},y_{i}\right)$ and $\left(x_{j},y_{j}\right)$, respectively, define the following variables:

- For the pairwise positions (i,j), *RSSDs* vector from all Aps is as follows:

(8) $${\Delta R}_{ij}=\left[{∆r}_{ij}^{1},\;{∆r}_{ij}^{2},\;\ldots ,{∆r}_{ij}^{m}\right],$$

where ${∆r}_{ij}^{k}=\left|r_{i}^{k}-r_{j}^{k}\right|$ indicates the RSSD of the k-th AP at pairwise positions (i,j).

- *PD* of the pairwise positions (i,j):

(9) $$d_{ij}^{p}=\sqrt{{\left(x_{i}-x_{j}\right)}^{2}+{\left(y_{i}-y_{j}\right)}^{2}},$$

Obviously, $d_{ij}^{p}=d_{ji}^{p}$.

- *FD* of the pairwise positions (i,j), which is the Euclidean distance of RSS vectors:

(10) $$d_{ij}^{f}=\sqrt{\sum_{k=1}^{m}{\left(∆r_{ij}^{k}\right)}^{2}},$$

It is noted that, from Equation (10), it can be seen that the contribution of different RSSDs to the discrimination capability of FDs is different. The bigger contribution of the RSSD $∆r_{ij}^{k}$ means the more suitable AP is selected.

In order to measure the whole discrimination capability of the FD against the PD in the localization area, the whole FD vector and PD vector are defined. For the fingerprint of n RPs in the fingerprint database, write the PD vector of all pairwise positions as follows:

(11) $$D^{p}=\left[d_{ij}^{p}\right],\left(0<i\leq n,j\leq n,i<j\right),$$

Similarly, the FD vector of all pairwise positions is as follows:

(12) $$D^{f}=\left[d_{ij}^{f}\right],0<i\leq n,j\leq n,i<j,$$

In this article, the relationships between vectors $D^{p}$ and $D^{f}$ are measured in correlation and consistency, which are used to measure the discrimination capacity of FD for PD.

## 4. Observation and Enhancement Policies for AP’s Discrimination Capability

In the current highly developed social environment of information technology, our smart devices can easily receive high-quality signals from many APs in public indoor environments.

These abundant APs are usually installed for the function of communication by different individuals, which are located at various positions and have various states, including being removed, impaired, unstable, etc. If there exist abnormal APs, it will inevitably lead to wrong FDs of pairwise positions, resulting in large localization errors. Therefore, utilizing such abundant existing APs to select suitable APs to improve the discrimination capability of FD has become an important factor affecting localization accuracy.

From the definition of FD, we have to emphasize AP’s contribution to RSS similarity. So, next, according to an in-depth analysis of the contribution of a single AP to the FD of a position pair, we aim to provide different AP RSS correction and selection ways, to greatly improve the discrimination capability of FD to a PD.

The strategy proposed in this section does not rely on increasing sample collection tasks or complex intelligent algorithms, but only on the presence of sufficient APs in indoor space. We aimed to improve FDDC by analyzing the physical performance of APs and RSS values.

### 4.1. Observations for Discrimination Capability

During the fingerprint-based indoor localization process, due to the influence of the distance, direction, and states of the APs, it often occurs that the identical RSSD implies a different PD between two positions.

#### 4.1.1. Diverse FDs’ Discrimination Capability Caused by Different Distance APs

**Figure 2:**
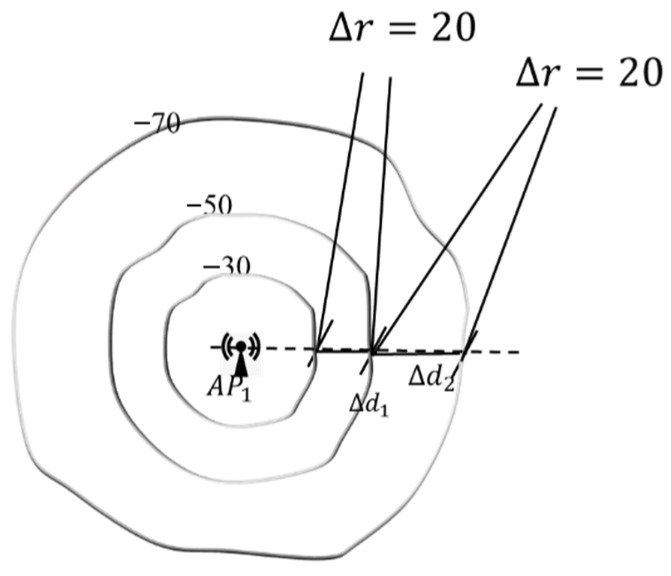
Discrimination capability diversity for APs with different distances.

It can be seen from Formula (2), RSS∝−log⁡(d), which indicates that $\frac{∆r}{∆d}\propto \frac{1}{d}$,where ∆r is the RSSD and ∆d denotes the corresponding distance difference. In other words, an identical ∆r means a larger PD difference ∆d at a farther position, or a smaller ∆d at a closer position. Similarly, an identical ∆d means a bigger RSSD ∆r at a closer position, or a smaller ∆r at a farther position. Figure 2 illustrates the RSS spatial distribution. It can be seen that the same RSSD of ∆r=20 corresponds to the different difference in PD of two pairwise positions, depending on the distances of the specific AP.

**Figure 3:**
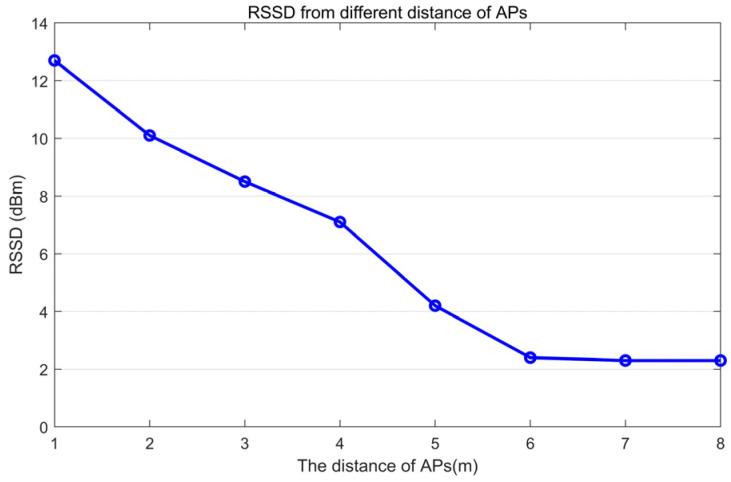
The different RSSDs for the same PD.

In order to investigate the impact of distance variation between AP and a location on the contribution of AP to RSS similarity, we observed the variation of RSSD with distance variation between AP and a location through a simple experiment. For two locations A and B with ∆d=1 m in our school library, connect BA and place an AP at different distances from A on the extension line on side A. Observe the RSSD of two locations A and B, as shown in the Figure 3.

As consistent with the above analysis, for the identical ∆d=1 m, the correspondence ∆r values are different and decrease with increasing distance. That is to say, APs with different distances have different RSSDs for the same two locations, so the contribution to RSS similarity is also different.

In a word, the PDs of pairwise positions indicated by RSSDs depend on the receivers’ distance, leading to varying discrimination capability for various positions.

#### 4.1.2. Diverse FDs’ Discrimination Capability Caused by APs with Different Directions

**Figure 4:**
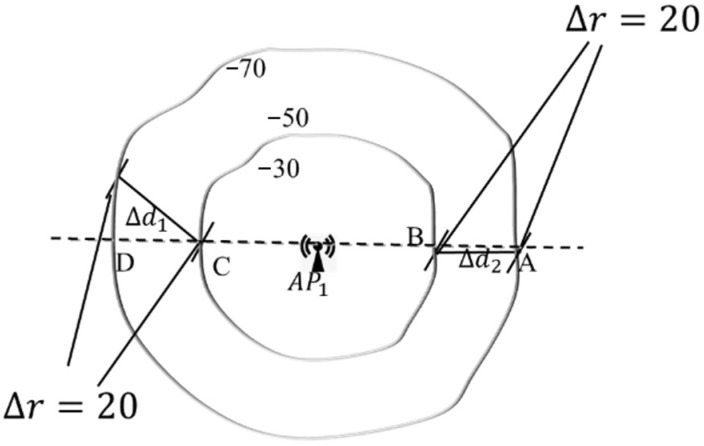
The discrimination capability diversity for APs with different directions.

Inherent constraints are constrained by the law of radio signal propagation; APs have diverse discrimination capabilities for different pairwise positions. That is, an identical ∆r can imply various PDs (see Figure 4). For any two positions, one is on the ring with RSS = −50 dBm, and the other is on the ring with RSS = −70 dBm, the value of RSSD ∆r is always 20, but the PD between them varies greatly. According to simple geometry, it’s easy to know that the PD of the position pair (A,B) is the smallest and (A,C) is the biggest. Meanwhile, the most accurate discrimination capability of RSSD ∆r=20 should be for position pair (A,B), which is related to the direction of the APs.

**Figure 5:**
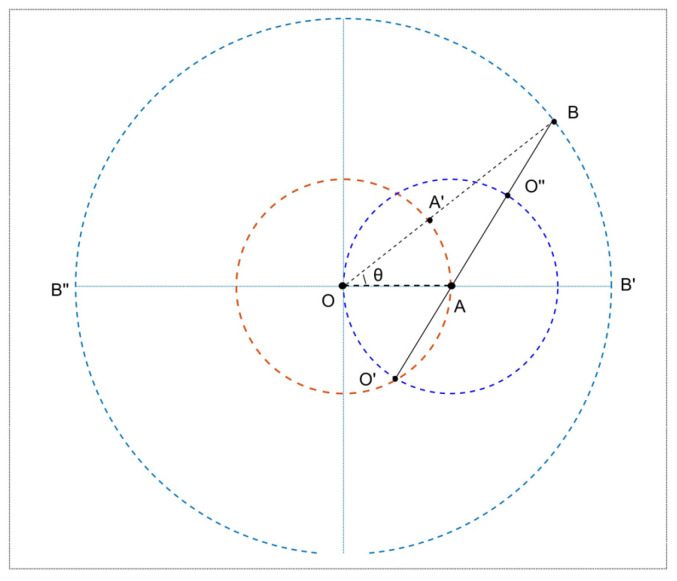
APs at different directions for a fixed position pair (A,B).

In other words, see Figure 5, assume that the pairwise position (A,B) is fixed, that is, $d_{AB}=d_{0}≔\Delta d$($d_{0}$ is a constant). Take position A as the center, and make a circle with a radius of $d_{OA}$ (smaller than $d_{0}$). Suppose an AP is located at *O*, then $d_{OA}$ is also a constant and $d_{OA}<d_{0}$, with O as the center, drawing two circles with a radius of $d_{OA}$ and $d_{OB}$, respectively. Connecting OB and OA, which will intersect with the two circumferences at points A′ and B′, respectively, and θ (0≤θ≤π) is the included angle between OA and OB.

It is obvious that θ changes with the location of the AP, but the correspondence between the RSSD $∆r_{AB}$ and radius difference $d_{OB}-d_{OA}$ remain unchanged, so set $d_{OB}-d_{OA}≔\Delta r$. Therefore, by studying the relationship between ∆r and θ, we can get that when the orientation of AP changes concerning the position pair, the corresponding discriminate capability of RSSD changes rules. For $∆AA^{\prime }B$, the following equation is gained:

(13) $$∆r=d_{0}-2d_{OA}sin\frac{\theta }{2},$$

We can obtain that the same value ∆d corresponds to different Δr, depending on the angle θ between line OA and OB, which means APs at different directions have different discrimination capabilities for the same position pair (A,B). As seen from Figure 5, when one AP is located at point $O^{\prime }$ and point $O^{\prime \prime }$, the discrimination capability of RSSD is optimal and poorest for position pair (A,B).

Remark that if $d_{OA}$ is a very small value, which means that the space between AP and point A is tiny enough, ∆d is about equal to the PD $d_{0}$. Then, the RSSD can characterize all PDs (A,B) well.

In a word, the PDs of the pairwise positions indicated by RSSDs depend on the receivers’ direction, leading to varying discrimination capability for various directions.

#### 4.1.3. Diverse FDs’ Discrimination Capability Caused by APs with Different Health States

In order to make the localization more accurate, in addition to the above two aspects, abnormal APs should not be ignored. Abnormal APs can be roughly divided into slight unhealthy (such as slight malfunction/objects blocking) and complete failure (such as broken/removed/newly emerged), which may occur in the online and offline stages. A slight abnormality means that the AP can transmit, but due to the device being impaired, the signal transmitted by the device is weak or unstable. Here, considering the complexity and variability of the indoor environment, for the obstruction of the new facilities or walls results in the weak signal of an AP at a specific location, we also treat the AP as slightly abnormal at the position. For complete failure/removed/newly emerged APs, although there are already works that can automatically enrich old fingerprint databases with new information [50], we are considering environments with rich APs and do not care about removing or installing individual APs. Hence, completely failed APs mean that the device cannot transmit signals at all in the offline and/or online stage.

**Figure 6:**
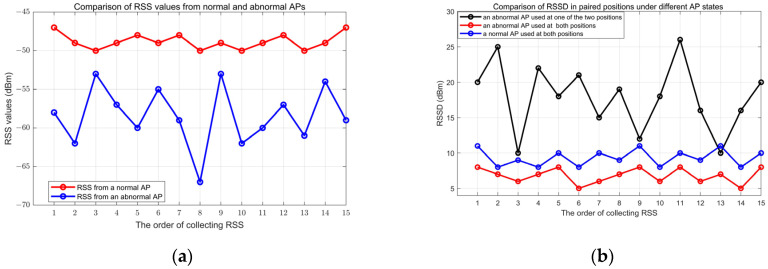
RSS value analysis. (**a**) The sampling point is 3 m away from AP. (**b**) RSSDs for position pair with 2.5 m.

In order to observe the effect of normal APs and abnormal APs, we carry out an experimental analysis of the RSS values of a specific position and the RSSD of a position pair. In this experiment, the position of the AP is known, and the power and signal lights of the AP are constantly being monitored. We use an iron box to rotate irregularly around the AP to simulate abnormal AP. First, for the sampling position that is fixed 3 m away from AP, we sample RSS values from a normal AP and an abnormal AP, respectively. The results are shown in Figure 6a. From the figure, it can be seen that the RSS values of the abnormal AP are obviously lower than the RSS value of the normal AP. In addition, the RSS values of the abnormal AP are extremely unstable compared to the values of normal AP. Second, for the sampling position pair with 2.5 m PD, we calculate the RSSD of the position pair (see Figure 6b). As seen, when the AP sampling at one position is normal and at the other position is abnormal, the RSSD is obviously larger than that of normal AP, and they are extremely unstable. When the AP sampling at both positions is abnormal, the RSSD is slightly smaller than that of normal AP and can be ignored when calculating FD.

In a word, the PDs of pairwise positions indicated by RSSDs are closely related to the AP’s state, leading to diverse FD’s discrimination capability for different healthy states.

### 4.2. FD’s Discrimination Capability Enhancement Policies

In this section, different discriminatory policies are discussed based on the above discussion and analysis. We first discuss the strategy to identify the abnormal Aps.

#### 4.2.1. Abnormal Aps Identification

In this section, we design two policies to reduce the impact of abnormal Aps, one for slight abnormality Aps and the other for complete failure/removed/newly emerged Aps.

Since the abundance of Aps in the indoor environment and the possibility of abnormal Aps, it is unnecessary and also unrealistic to utilize the RSSDs of all Aps equally. Whether for the fingerprint in the fingerprint database or online collected the RSS of TP, set a threshold for RSS values to exclude Aps with weak signal strength.

For position i, the RSS vector collected from m Aps is $R_{i}=\left[r_{i}^{1},r_{i}^{2},\ldots ,r_{i}^{m}\right]$, the RSS value $r_{i}^{k}$ is defined as follows:

(14) $$r_{i}^{k}=\left\{\begin{array}{cc} r_{i}^{k} & r_{i}^{k}\geq R\\ -100 & r_{i}^{k}<R \end{array}\right.$$

where R is a threshold, only the RSS values of Aps with RSS values greater than R will be used to participate in the following steps, otherwise, RSS values will be set to 0. Through this method, slightly abnormal Aps with small RSS values (<R) in the environment can be eliminated. Furthermore, from Equations (8) and (10), we can find that the component ${∆r}_{ij}^{k}$ of RSSD directly impacts the FD $d_{ij}^{f}$. An AP is normal at one position but is a complete failure or removed or newly emerged at the other position, which can generate a significant ${∆r}_{ij}^{k}$ and affect the discrimination capability of FDs. To avoid this situation, make the following rule for ${∆r}_{ij}^{k}$:

(15) $${∆r}_{ij}^{k}=\left\{\begin{array}{cc} \left|r_{i}^{k}-r_{j}^{k}\right| & r_{i}^{k}\neq -100\;and\;r_{j}^{k}\neq -100\\ 0 & r_{i}^{k}=-100\;or\;r_{j}^{k}=-100 \end{array}\right.,$$

We assume that there always exist rich Aps in indoor scenes, and then through the above policies, abnormal Aps, especially the complete failure or removed or newly emerged Aps in the environment, can be eliminated almost.

#### 4.2.2. Discrimination Correction Quantity

In indoor environments, due to signal reflection and refraction, or multipath fading and indoor noise, signal attenuation and RSS fluctuation are severe. Especially, they are closely related to the current environment. Therefore, we hope to adjust the RSS values generated by each AP at each location to weaken the impact of the environment. More generally speaking, the closer Aps are less affected by environmental uncertainty, and the correction rule is to emphasize closer Aps.

Here, we define a correction factor to quantitatively differentiate each AP for a specific location. According to the LDPL model (2) and the estimation of PDs between AP and a position, for the *k*-th AP to the *i*-th position, the correction factor is calculated as follows:

(16) $$\gamma_{i}^{k}=\frac{1}{{\hat{d}}_{i}^{k}}=10^{\frac{r_{i}^{k}-P_{R}(d_{0})}{10n}}$$

where ${\hat{d}}_{i}^{k}$ is the estimated distance between the *k*-th AP and the *i*-th position.

Furthermore, to reduce the strong signal influence, we introduce the way in [17] to redefine the correction factor as follows:

(17) $$\gamma_{i}^{k}=\left\{\begin{array}{c} 10^{\frac{r_{i}^{k}-P_{R}(d_{0})}{10n}}\;\;\;\;\;\;\;\;\;\;\;\;\;\;\;\;\;if\;r_{i}^{k}\leq r_{0}\\ \frac{1}{a}{\left(1+e^{-2\left(\frac{r_{i}^{k}+100}{10}-c\right)}\right)}^{-1}\;\;\;\;otherwise \end{array}\right.,$$

where $r_{0}$ is a flexible value, it can be given by empirical, for example, −50 dBm, a and c are constant parameters, which can be determined based on n such that $\gamma_{i}^{k}$ is continuous at $r_{0}$.

From Formula (17), because the reciprocal of PD is consistent with the monotonicity of the LDPL model, the closer the AP is, the greater the value of the correction factor. Therefore, the correction quantity is defined as follows:

(18) $$\varphi_{i}^{k}=\Phi \cdot \gamma_{i}^{k}$$

where Φ is a flexible empirical value according to the environment conditions, usually between 3 dBm and 6 dBm, approximately equal to the value of signal fluctuation at a position.

#### 4.2.3. Priority Weight

From Formula (13) in Section 4.1.2, we can gain a rule: for Aps in different directions, the smaller the angle θ is, the larger the RSSD Δr is, which means that the direction in which the AP generates a large RSSD is very advantageous for positions A and B. In other words, the Aps who get the larger RSSD have a greater contribution to FD’s discrimination capability. Here, we want to emphasize the importance of this AP by assigning higher priority weight to the RSSD.

Therefore, we grant each RSSD of each pairwise position from each AP a priority weight $\tau_{ij}^{k}$ as follows, which determines the priority and contribution of its participation in FD (see Equation (19)). As seen, the larger RSSD corresponds to a higher priority weight.

(19) $$\lambda_{ij}^{k}=\frac{1}{1+e^{-∆r_{ij}^{k}}},$$

It is clear that $\lambda_{ij}^{k}$ is the sigmoid function of $∆r_{ij}^{k}$, so adding $\lambda_{ij}^{k}$ to RSSD will not have a significant impact on the value of FD, avoiding affecting localization accuracy.

Note that abnormal Aps often generate large RSSD, and in this case, adding priority weights can increase the error. Therefore, priority weights need to be used after removing abnormal Aps.

Overall, through the above-provided discrimination capability improved policies, the Aps with larger contributions to FDs’ discrimination capability will be involved in the calculation of FDs, and the more suitable the Aps, the higher the degree of participation. Hence, FDs with strong discrimination capability to PDs are obtained, providing a guaranteed prerequisite for relevant fingerprint matching algorithms.

## 5. WKNN and FDDC-WKNN Algorithm

The K-nearest neighbor (KNN) algorithm is a simple and practical machine learning algorithm that is one of the most widely used fingerprint matching algorithms. By setting different weights to the K nearest neighbors, WKNN often performs better in localization performance.

Essentially, both KNN and WKNN obtain similar distances through RSS similarity. Thereby, we expect the above-provided FDs discrimination capability improved policies can be combined with WKNN to improve localization performance, namely FDDC-WKNN.

Moreover, for different TPs in a large-scale indoor space, it is often necessary to select different subsets of Aps. Hence, the FDDC-WKNN algorithm process also has the function of dynamically selecting AP subsets.

### 5.1. The Idea of KNN and WKNN Algorithms

The idea of the KNN algorithm is to select the positions of K fingerprints closest to the current positional fingerprint to estimate the current position, which is simple, intuitive, and effective. KNN is one of the algorithms that use FD of pairwise positions to discriminate PD between them.

We assume that there are n RPs in an indoor area, and n fingerprints were collected in the offline phase, which is denoted as ${\left[F_{1},F_{2},\cdots ,F_{n}\right]}^{T}$, where $F_{i}=[x_{i},y_{i},R_{i}]$, see Equations (6) and (7). In the online phase, the RSS vector of a TP is $R_{t}$. First, calculate all FDs $d_{ti}^{f}(0<i\leq n)$ from position S to RPs; second, select *K* nearest RPs based on all FDs; finally, use the following Formula (20) to get the estimation value of S coordinates:

(20) $$S=:\left(\hat{x},\hat{y}\right)=\frac{1}{K}\sum_{i=1}^{K}\left(x_{i},y_{i}\right)$$

The above formula indicates that the KNN algorithm sets the same weights to all K nearest neighbors. However, according to the propagation characteristics of indoor signals, it is known that the farther the distance, the stronger the signal fluctuation. For this reason, the WKNN algorithm sets different weights based on FD to emphasize that close neighbors contribute ability more, the estimation value of S coordinates can be expressed as follows:

(21) $$S=:\left(\hat{x},\hat{y}\right)=\frac{1}{\sum_{i=1}^{K}w_{i}}\sum_{i=1}^{K}\left(w_{i}x_{i},{w_{i}y}_{i}\right)$$

where $w_{i}$ is the weight related to distance. In this article we adopt the WKNN algorithm to be the indoor fingerprint algorithm, and the weights are given in the following formula.

(22) wi=1dtif∑i=1K1dtif

Note that: Different K values mean that the number of nearest RPs selected is different, which can affect the accuracy of localization. The optimal K value is often gained from experience or practice.

### 5.2. The FDDC- WKNN Algorithms

Taking into account all the strategies and WKNN ideas discussed above, a uniform WKNN solution based on improving FD’s discrimination capability will be presented in this section, that is FDDC-WKNN. The presence of a large amount of abundant Aps in the indoor environment is a prerequisite for this solution.

For an indoor area, given that there are $\tilde{N}$ RPs and $\tilde{M}$ Aps and fingerprint databases have been built on the Server. In the online phase, when we have the RSS vectors ${\tilde{R}}_{t}$ at a TP, send them to the server, and want to get the TP location $\left(\hat{x},\hat{y}\right)$. Based on the policies aforementioned, the algorithm steps on the server side are given as follows:

Step1. Set a threshold R, modify the RSS value in ${\tilde{R}}_{t}$ and the fingerprint data in the dataset by Formula (14). First, select a subset of Aps with a quantity of M: sort all RSS values > −100 ${\tilde{R}}_{t}$ and select the corresponding top M Aps. Then, select RPs in the TP neighboring domain: check if all RSS of one RP under the above AP subset is greater than −100; if so, then it is considered that the RP belongs to the TP neighboring domain. Assume that the number of RPs belonging to the TP neighboring domain is N, and the RSS vector of TP under this AP subset is denoted as $R_{t}$.

Step2. Combine $R_{t}$ and the RSS matrix of the N RPs to a new matrix $F^{\prime }$ as follows, where the first row of $F^{\prime }$ is RSS vector $R_{t}$ at TP, and the other N rows are the RSS matrix at RPs.

(23) $$F^{\prime }=\left[\begin{array}{cc} \begin{array}{cc} r_{t}^{1} & r_{t}^{2}\\ r_{1}^{1} & r_{1}^{2} \end{array} & \begin{array}{cc} \cdots & r_{t}^{M}\\ \cdots & r_{1}^{M} \end{array}\\ \begin{array}{cc} \begin{array}{c} r_{2}^{1}\\ \vdots \\ r_{N}^{1} \end{array} & \begin{array}{c} r_{2}^{2}\\ \vdots \\ r_{N}^{2} \end{array} \end{array} & \begin{array}{cc} \begin{array}{c} \cdots \\ \ddots \\ \cdots \end{array} & \begin{array}{c} r_{2}^{M}\\ \vdots \\ r_{N}^{M} \end{array} \end{array} \end{array}\right]:={\left[\begin{array}{ccc} \begin{array}{cc} R_{t} & R_{1} \end{array} & R_{2} & \begin{array}{cc} \cdots & R_{N} \end{array} \end{array}\right]}^{T},$$

Step3. Calculate the discrimination factor $\gamma_{i}^{k}$ for each RSS value and gain the discrimination correction quantity matrix Γ as follows:

(24) $$\Gamma ={\left[\begin{array}{ccc} \begin{array}{cc} \Gamma_{t} & \Gamma_{1} \end{array} & \Gamma_{2} & \begin{array}{cc} \cdots & \Gamma_{N} \end{array} \end{array}\right]}^{T},$$

where

(25) $$\Gamma_{i}={\left[\begin{array}{ccc} \begin{array}{cc} \varphi_{i}^{1} & \varphi_{i}^{2} \end{array} & \cdots & \varphi_{i}^{M} \end{array}\right]}^{T},(i=t\;or\;1\leq i\leq N),\;\varphi_{i}^{k}=\Phi \cdot \gamma_{i}^{k}$$

Then, we calculate modified RSS data by $F^{\prime }$ plus discrimination correction quantity matrix Γ, see Equation (26). Here, ${\tilde{R}}_{1}$ can be seen as the RSS value corrected by the discrimination factor.

(26) $$F^{\prime }+\Gamma ={\left[\begin{array}{ccc} \begin{array}{cc} R_{t}+\Gamma_{t} & {R_{1}+\Gamma }_{1} \end{array} & R_{2}+\Gamma_{2} & \begin{array}{cc} \cdots & R_{N}+\Gamma_{N} \end{array} \end{array}\right]}^{T}≔{\left[\begin{array}{ccc} \begin{array}{cc} {\tilde{R}}_{t} & {\tilde{R}}_{1} \end{array} & {\tilde{R}}_{2} & \begin{array}{cc} \cdots & {\tilde{R}}_{N} \end{array} \end{array}\right]}^{T}≔\tilde{F},$$

where

(27) $${\tilde{R}}_{i}=(r_{i}^{1}+\varphi_{i}^{1},r_{i}^{2}+\varphi_{i}^{2},\cdots ,r_{i}^{M}+\varphi_{i}^{M})(i=t\;or\;1\leq i\leq N),$$

Step 4. Calculate the RSSD of the TP for each RP, that is, subtract each remaining row of the matrix $\tilde{F}$ from the first row to obtain each RSSD vector $∆R_{ti}(1\leq i\leq N)$. Then, an RSSD matrix is gained as follows:

(28) $${∆R=\left[\begin{array}{ccc} \begin{array}{cc} ∆R_{t1} & ∆R_{t2} \end{array} & \cdots & ∆R_{tN} \end{array}\right]}^{T},$$

where

(29) $$∆R_{ti}=\left|{\tilde{R}}_{t}-{\tilde{R}}_{i}\right|\;(1\leq i\leq N),$$

Use priority weight Formula (19) to calculate all priority weight of $∆R_{ti}(1\leq i\leq N)$, we can get the following priority weight matrix Λ.

(30) $$\Lambda ={\left[\begin{array}{ccc} \begin{array}{cc} \Lambda_{t1} & \Lambda_{t2} \end{array} & \cdots & \Lambda_{tN} \end{array}\right]}^{T},$$

where $\Lambda_{ti}={\left[\begin{array}{ccc} \begin{array}{cc} \lambda_{ti}^{1} & \lambda_{ti}^{2} \end{array} & \cdots & \lambda_{ti}^{M} \end{array}\right]}^{T},(1\leq i\leq N)$.

Step5. Calculate the Hadamard product of Λ and ∆R, that is multiply the corresponding elements of Λ and ∆R as Equation (31). Then, we get the weighted RSSD $∆\tilde{R}$ by priority weight.

(31) $$\Lambda ⊙∆R=≔{\left[\begin{array}{ccc} \begin{array}{cc} ∆{\tilde{R}}_{t1} & ∆{\tilde{R}}_{t2} \end{array} & \cdots & ∆{\tilde{R}}_{tN} \end{array}\right]}^{T}≔∆\tilde{R},$$

where

(32) $$∆{\tilde{R}}_{ti}=\Lambda_{ti}∆R_{ti}(1\leq i\leq N),$$

Step 6. Calculate the FD of TP to each RP by Formula (10) to obtain an FD sequence $D_{t}^{f}$ and express it by the following Equation (33):

(33) $$D_{t}^{f}=\left(\begin{array}{cc} \begin{array}{cc} d_{t1}^{f} & d_{t2}^{f} \end{array} & \begin{array}{cc} \cdots & d_{tN}^{f} \end{array} \end{array}\right),$$

where

(34) $$d_{ti}^{f}=\sqrt{\sum_{k=1}^{M}{\left(\lambda_{ti}^{k}\left|\varphi_{t}^{k}r_{t}^{k}-\varphi_{i}^{k}r_{i}^{k}\right|\right)}^{2},}$$

Step7. Select the K RPs corresponding to the smallest K elements in sequence $D_{t}^{f}$, and the positions of these K RPs are the K positions closest to TP. Then, calculate the weight of each RP according to Formula (22). Finally, according to Formula (21), we can get the location $\left(\hat{x},\hat{y}\right)$ of TP.

In addition, in order to obtain the correlation and consistency between PD and FD in subsequent experiments, we need to calculate the PD of TP to each RP by Formula (9) to obtain an PD sequence $D_{t}^{p}$ and express it as follows:

(35) $$D_{t}^{p}=\left(\begin{array}{cc} \begin{array}{cc} d_{t1}^{p} & d_{t2}^{p} \end{array} & \begin{array}{cc} \cdots & d_{tN}^{p} \end{array} \end{array}\right),$$

where

(36) $$d_{ti}^{p}=\sqrt{{\left(x_{t}-x_{i}\right)}^{2}+{\left(y_{t}-y_{i}\right)}^{2}}$$

Compared to the WKNN algorithm, with a time complexity of O(MN+NK+K), the FDDC-WKNN algorithm requires additional steps such as selecting subsets of Aps (step 1), calculating correction quantity (step 3), and calculating priority weight (step 4). With a time complexity of $O(\tilde{M}+\tilde{M}\tilde{N}+N+MN+NK+K)$, the time complexity has increased by $O(\tilde{M}+\tilde{M}\tilde{N}+N)$. During the fingerprint matching process, the sizes of $\tilde{M}$ and N are both limited, ranging from tens to hundreds, so the impact on time efficiency is linear, and the FDDC algorithm has high time performance.

## 6. Simulation and Experiments

In this section, we will evaluate the policies and algorithms proposed in this article by conducting some experiments in two scenes: simulation and real environments. All simulations and experiments were conducted on a personal laptop using MATLAB V9.5. The computer is the 64-bit operating system based on x64 processors, and the hardware configuration is an Intel(R) Core(TM) i7-8550U CPU with 16.0 GB of RAM.

In particular, our technology is suitable for having a large number of Aps in both scenes, and there are phenomena such as Aps being damaged, malfunctioning, removed, etc.

### 6.1. Scenes Setup and Some Parameter Presets

First, the environment settings, fingerprint database construction, and some presets in both simulated and real scenarios were introduced.

#### 6.1.1. Introduce for Experimental Scene

- Scene 1: simulation scene

**Figure 7:**
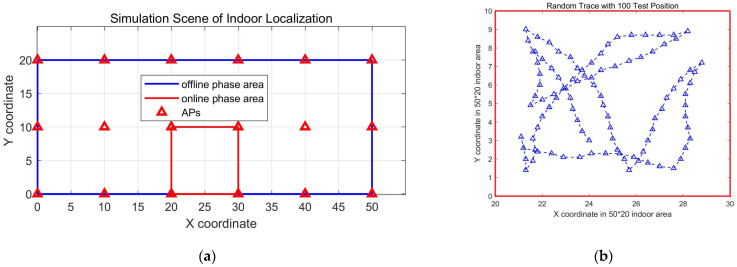
Simulation scene of indoor localization. (**a**) Details of the simulation area; (**b**) Random trace with 100 TPs.

To simulate large-scale indoor space with rich Aps, we simulated a 50 m×20 m sized indoor area with 18 Aps, and these Aps are placed on grid points every 10 m in the simulation area, as depicted in Figure 7a. In our simulation process, RSS data are generated by ray tracing technology based on the LDPL model, which considers multiple propagation paths between the transmitting and receiving points, analyzes the signals of each path separately, and then superimposes to obtain the RSS value on each receiving point. Here, we mainly consider the issue of wall reflection paths and signal attenuation. The simulation was completed in MATLAB. Initially, we generated a simulation fingerprint dataset at position with an interval of 0.1 m. Then, we can get any offline fingerprint database from the dataset as needed. For example, an offline fingerprint database with an RPs interval of 1 m only needs to take values every 10 position points in the dataset, that is, 10×0.1=1 m, and so on.

In our following simulation experiment, the offline fingerprint database is from the above fingerprints dataset by setting the RPs interval of the offline database to 1 m, that is, there are 51×21=1071 RPs in the simulation area, and there are a total of RSS values from 18 Aps at each RP.

In the online phase, we simulated a target moving in a 100 square meter room in the simulation area (see the red rectangle in Figure 7a) and obtained a trace with 100 trajectory points, as well as RSS on each trajectory point (see Figure 7b). Meanwhile, we simulated two abnormal Aps, one of which was removed or broken, and the other had a minor malfunction. These 100 trajectory points are used as TPs for the indoor localization algorithm. In order to simulate people’s living habits, the movement trajectory moves in space within 1 m of the boundary.

- Scene 2: real library scene

**Figure 8:**
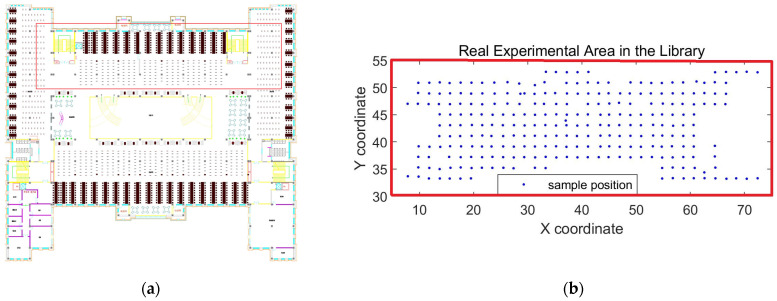
Real scene of indoor localization. (**a**) The plan of the second floor in the school library. (**b**) Real experiment area with APs.

The real experimental scene was on the second floor of the school library at Dalian University, and its plan is shown in Figure 8a. The central position of this floor is the vacant part on the first floor, surrounded by an open bookshelf reading area, including a large number of open bookshelves and reading tables and chairs. The experiment area is located on the north side of the floor with about 1200 m^2^, in the red rectangle area of Figure 8a, and Figure 8b is the enlarged experiment area. As can be seen, there are 261 RPs at an interval of 2 m. In addition, there are a total of 83 Aps that can work in the experimental area.

In the offline phase, we used an indoor positioning system that includes both server and mobile devices, which was developed and implemented in collaboration with a company. The server is deployed on a dual 2U ThinkServer, and the mobile APP is installed on the HUWEI P9 smartphone. Before building the fingerprint database, it is necessary to load the building structure plan into the server, and the real RPs can be marked in the plan, as shown in Figure 8. Then, the handheld intelligent mobile terminal can be used for RSS collection in real RPs. At each RP, 15 RSS values for each received AP of 83 APs were collected, and the average value as the final RSS value was saved. In the online phase, to reduce the workload of data sampling, we randomly selected 50 points from these 261 to be TPs and set 8 abnormal APs with 4 APs removed or breakdowns and 4 APs with slight abnormality.

Overall, in order to have a clearer understanding of the two scenes, the setups for both scenes are shown in Table 1.

#### 6.1.2. Parameters Presetting

In the proposed algorithm, many parameters need to be set to experiment. Here, considering the environments of two scenes, the parameters are determined based on experience or specific conditions, respectively, as listed in Table 2.

Due to the RSS being sampled directly in the real scene, the first three parameters are not required to be set.

### 6.2. Simulation and Analysis

Aiming to measure whether the proposed policies can improve the whole discrimination capability of FD to PD, we conducted some experiments to verify the correlation and consistency changes between FD and PD before and after using FDDC-WKNN. For the simulation environment, we first verified the effect of proposed policies on the discrimination capability of FD to PD. Then, for a random trace with 100 positions, we find the optimal value of K, and with the optimal K, we simulate the localization positions and derive the CDF curves to demonstrate the localization performance.

For the real scene in the Dalian University library, we also find the optimal value of K for 50 random TPs, and with the optimal K, we gain the localization results similarly.

#### 6.2.1. Discrimination Capability of FD to PD

**Figure 9:**
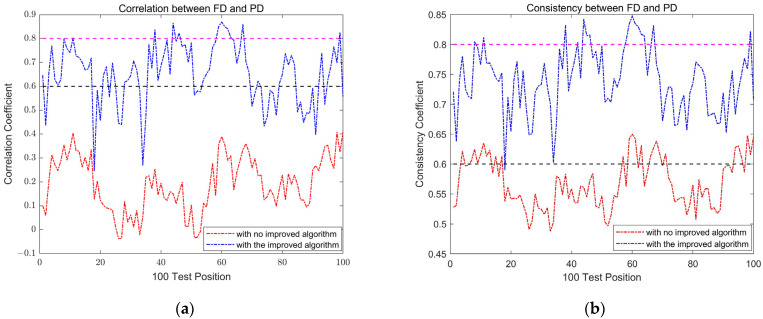
Correlation and consistency between PD and FD. (**a**) Correlation coefficient before and after using the proposed policies. (**b**) Consistency coefficient before and after using the proposed policies.

By calculating the PD sequence $D_{t}^{p}$ using Equation (35) and the FD sequence $D_{t}^{f}$ using Equation (33) between 100 TPs and 81 RPs in the localization area, we gain the correlation coefficient and consistency coefficient between each TP and RP. From the results shown in Figure 9, we can see that both the correlation coefficient and the consistency coefficient have greatly improved. After implementing the policies proposed in this article, strong correlation increased from 0 to 76%, and consistency increased from 26% to 99%, which indicates that the discrimination capability of FD to PD has been greatly improved.

To further examine the impact of abnormal AP on FDs’ discrimination capability, we repeated the experiment of calculating $D_{t}^{p}$ and $D_{t}^{f}$ under diverse abnormal AP conditions to obtain the correlation coefficient and consistency coefficient between PD and FD. For clarity, the experimental conditions are recorded as the following situations, and the results before and after implementing FDDC_WKNN are displayed in Table 3.

Ⅰ: All APs are in a normal state.

Ⅱ: There exists one AP with a slight abnormality.

Ⅲ: There exists one AP with a complete failure.

From the results in Table 2, it can be seen that the presence of completely failed APs in the localization area has the greatest impact on the correlation and consistency between FD and PD. Moreover, regardless of the conditions, the policies proposed in this article have improved the correlation and consistency between FD and PD to varying degrees.

#### 6.2.2. Setting of K

In the same conditions of localization area, different K values in FDDC-WKNN can lead to different localization accuracy. We assumed that the K value with the lowest average errors was optimal. The optimal K value is usually not fixed and varies depending on the environment. We compared the average errors under different K values for both simulation and real scenes to obtain the corresponding optimal K value.

**Figure 10:**
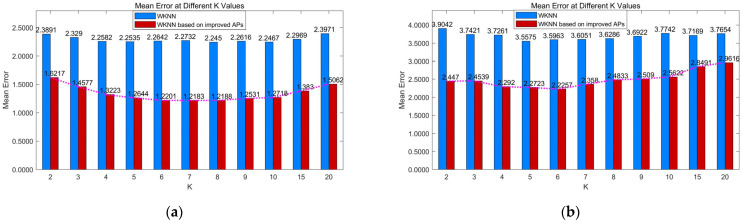
Average localization errors under different K values. (**a**) In the simulation scene. (**b**) In the real library scene.

As shown in Figure 10, the optimal K value is 7 in the simulation scene and 6 in the real scene. However, we found that the average localization errors did not change significantly with different K values. Although our policies have greatly improved the FD’s capability to discriminate PD, the optimal K value is only reduced from 7 to 6.

#### 6.2.3. Localization Examination by FDDC-WKNN under the Optimal K

**Figure 11:**
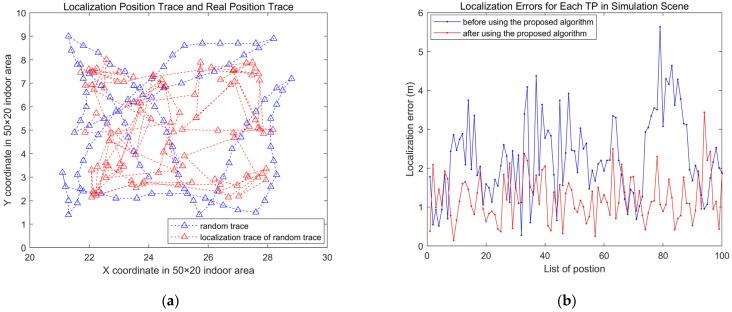
Localization results applying FDDC-WKNN for simulation scene with 100 TPs. (**a**) The original position and localization results of TPs. (**b**) Localization errors of each TP.

**Figure 12:**
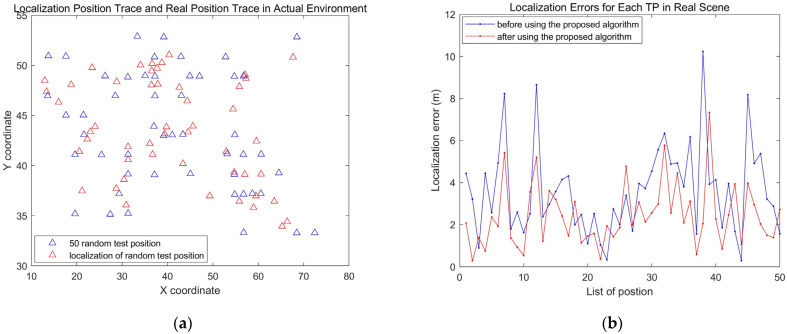
Localization results applying FDDC-WKNN for the real library scene with 50 TPs. (**a**) The original position and localization results of TPs. (**b**) Localization errors of each TP.

For K=7 in the simulation scene with 100 TPs and K=6 in the real library scene with 50 TPs, we apply the FDDC-WKNN algorithm, and the localization results are demonstrated in Figure 11 and Figure 12, respectively.

In the simulation scene, Figure 11a shows the locations of TPs and the estimated locations of TPs obtained based on the FDDC-WKNN algorithm. It can be seen from Figure 11b that before using the FDDC-WKNN algorithm, due to the influence of abnormal Aps, the localization errors were large and fluctuated greatly, while the localization errors have significantly decreased and are stable after using the FDDC-WKNN algorithm.

In the real library scene, the localization results are basically consistent with the simulation scene (see Figure 12). Figure 12a shows the locations of TPs and the estimated locations of TPs obtained based on the FDDC-WKNN algorithm. Due to the complexity and variability of the real environment, the localization errors are larger and the error fluctuation is also greater (see Figure 12b).

**Figure 13:**
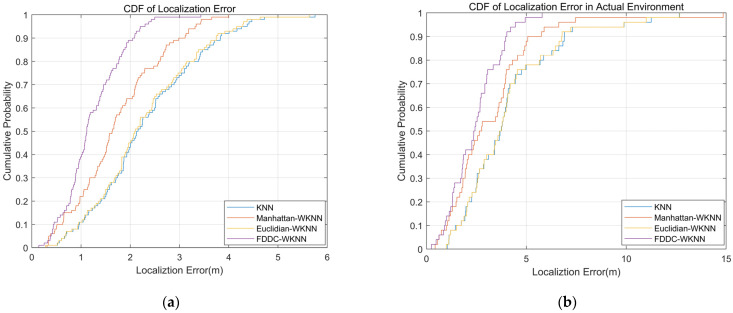
Comparison of the CDF between FDDC_WKNN and other algorithms. (**a**) In the simulation scene. (**b**) In the real library scene.

Moreover, Figure 13 presents a comparison of the CDF between FDDC-WKNN and other algorithms, including KNN, Euclidian-WKNN, Manhattan-WKNN, and KNN, which are related popular algorithms involved in fingerprint-base localization. The FDDC-WKNN algorithm, which considers AP contribution issues, has better accuracy and stability than other algorithms.

In order to more accurately demonstrate the localization errors of several algorithms, Table 3 presents the mean errors of these several algorithms. From Table 4, it can be seen that the mean errors have been reduced from 2.2732 m to 1.2290 m and from 4.0489 m to 2.4320 m in both the simulation scene and the real library scene, respectively.

In summary, we have examined the WKNN algorithm based on the improved FDs’ discrimination capability proposed in this article through multi-directional experiments. All experimental results indicate that the proposed approach can improve FD’s capability to discriminate PD in environments with abnormal APs, thereby reducing localization errors and improving localization accuracy. In addition, the proposed algorithm performs stably in the presence of interference in the environment, proving its robustness.

## 7. Conclusions

In this article, by defining a threshold, a correct quantity, and a priority weight, the capability of FD to discriminate PD is greatly improved for complexity and AP-rich indoor environments. Furthermore, combined with WKNN, a new indoor fingerprint localization technique referred to as FDDC-WKNN was provided. The FDDC-WKNN algorithm is particularly suitable for large-scale indoor scenarios with ubiquitous uncontrollable APs and has a stable and robust localization performance.

However, the large-scale indoor scenes considered in this article are relatively stable; for indoor scenes with frequent changes, corresponding updates to the fingerprint dataset are required to cooperate, which will bring a heavy workload.

In addition, during large-scale indoor movement, the selected AP subset also changes dynamically due to the different positioning target positions. Therefore, it is necessary to select the AP subset for each TP. In order to improve time efficiency, the initial AP selection in this article is only filtered through a threshold. In fact, before conducting RSS similarity calculations, a suitable subset of AP can be further screened, which will be a future research direction.

The discrimination capability of FD to PD is the key foundation for most indoor localization methods based on FD, which is only applied and validated in the WKNN algorithm in this article. Next, the algorithm will be implemented, verified, and improved using different indoor localization techniques.

Also, improving the accuracy of localization with the other various intelligent algorithms and auxiliary facilities, studying different strategies based on various environmental requirements, and so on are the future study directions.

## Figures and Tables

**Table 1:** Setups for two scene.

	Setups	Sample Dimensions	Experiment Dimensions	Number of APs	APs’ Positions	Abnormal APs	Number of TPs
Scenes							
**Scene 1**		2500 m^2^	100 m^2^	18	Grid points/10 m	2	100
**Scene 2**		1200 m^2^	1200 m^2^	83	Unknown	8	50

**Table 2:** Presets for parameters in the experiment.

Parameters	Scene 1	Scene 2
Path loss exponent n	2	/
Reference distance $\;d_{0}$	1	/
Power at $\;d_{0}$	−39	/
Parameters $r_{0},a,c$ in the impact factor	−55, 3.4, 4.288	−55, 3.4, 4.288
Threshold R	−70	−90
Fluctuating quantity Φ	3	5

**Table 3:** The proportion of strong correlation and high consistency between PDs and FDs before and after implementing the proposed policies.

	Situations	I		II		III		II and III	
Correlations									
**Strong Correlation**		16%	91%	16%	89%	0	78%	0	76%
**High Consistency**		72%	99%	78%	99%	27%	99%	26%	99%

**Table 4:** The mean error of several algorithms.

	Algorithms	FDDC-WKNN	Euclidian-WKNN	Manhattan-WKNN	KNN
Scenes					
**S** **cene** **1**		1.2290	2.2732	1.7332	2.3221
**S** **cene** **2**		2.4320	4.0489	3.2180	4.0755

## Data Availability

For the purpose of research, scholars can contact Baofeng Wang to obtain the data presented in this study.
